# Single dose GLP toxicity and biodistribution study of a conditionally replicative adenovirus vector, CRAd-S-pk7, administered by intracerebral injection to Syrian hamsters

**DOI:** 10.1186/s12967-016-0895-8

**Published:** 2016-05-16

**Authors:** Julius Woongki Kim, Brenda Auffinger, Drew A. Spencer, Jason Miska, Alan L. Chang, Joshua Robert Kane, Jacob S. Young, Deepak Kanojia, Jian Qiao, Jill F. Mann, Lingjiao Zhang, Meijing Wu, Atique U. Ahmed, Karen S. Aboody, Theresa V. Strong, Charles D. Hébert, Maciej S. Lesniak

**Affiliations:** Department of Neurological Surgery, Northwestern University Feinberg School of Medicine, 676 N. St Clair St, Suite 2210, Chicago, IL 60611 USA; City of Hope, Duarte, CA USA; University of Alabama at Birmingham, Birmingham, AL USA; Southern Research Institute, Birmingham, AL USA

**Keywords:** CRAd-S-pk7, Conditionally replicative adenovirus, Oncolytic adenovirus, Toxicity, Biodistribution, Immune response

## Abstract

**Background:**

CRAd-S-pk7 is a conditionally replicative oncolytic adenoviral vector that contains a survivin promoter and a pk7 fiber modification that confer tumor-specific transcriptional targeting and preferential replication in glioma while sparing the surrounding normal brain parenchyma.

**Methods:**

This IND-enabling study performed under GLP conditions evaluated the toxicity and biodistribution of CRAd-S-pk7 administered as a single intracerebral dose to Syrian hamsters, a permissive model of adenoviral replication. Two hundred and forty animals were stereotactically administered either vehicle (*n* = 60) or CRAd-S-pk7 at 2.5 × 10^7^, 2.5 × 10^8^, or 2.5 × 10^9^ viral particles (vp)/animal (each *n* = 60) on day 1. The animals were closely monitored for toxicology evaluation, assessment of viral distribution, and immunogenicity of CRAd-S-pk7.

**Results:**

Changes in hematology, clinical chemistry, and coagulation parameters were minor and transient, and consistent with the inflammatory changes observed microscopically. These changes were considered to be of little toxicological significance. The vector remained localized primarily in the brain and to some degree in the tissues at the incision site. Low levels of vector DNA were detected in other tissues in a few animals suggesting systemic circulation of the virus. Viral DNA was detected in brains of hamsters for up to 62 days. However, microscopic changes and virus-related toxicity to the central nervous system were considered minor and decreased in incidence and severity over time. Such changes are not uncommon in studies using adenoviral vectors.

**Conclusion:**

This study provides safety and toxicology data justifying a clinical trial of CRAd-S-pk7 loaded in FDA-approved HB1.F3.CD neural stem cell carriers administered at the tumor resection bed in humans with recurrent malignant glioma.

**Electronic supplementary material:**

The online version of this article (doi:10.1186/s12967-016-0895-8) contains supplementary material, which is available to authorized users.

## Background

Despite recent advances in glioma treatments such as surgical resection, chemotherapies, and novel drug therapies, the survival rate of patients with advanced-grade glioma remains dismal [1]. For that matter, the desperate clinical context of high-grade glioma warrants the present development of novel strategies with the potential for direct patient impact. An emerging, highly promising strategy for the treatment of glioma is the delivery of specific therapeutic agent(s) to tumor areas such as oncolytic viruses, aptly termed, oncolytic virotherapy [1–4].

Among the oncolytic viruses, human adenovirus serotype 5 (Ad5) is the most commonly used due to its well-studied biology and flexibility for genetic modifications [2, 3]. By taking advantage of these characteristics, a conditionally replicating oncolytic adenovirus, CRAd-S-pk7, has been generated [5]. This viral vector infects cells by binding to anionic cell surface proteins through seven lysine residues (pk7) on the adenoviral fiber [6, 7] and subsequently initiates replication by way of *E1* gene expression under the control of the tumor-specific promoter survivin (S) [5]. This glioma selective oncolytic agent, CRAd-S-pk7, was shown to efficiently lyse glioma cells while sparing non-neoplastic cells [8, 9]. However, the efficacy of CRAd-S-pk7 in the clinical setting can be nullified via both immune surveillance and pre-existing neutralizing antibodies against Ad5 [8, 9]. Moreover, the biodistribution of oncolytic viruses (OVs) may be limited to the outer borders of a solid tumor mass with robust vascular supply, which often results in inconsistent viral penetration to the center of the tumor mass [8–10].

To surmount these problems, we employed a cell carrier approach with neural stem cells (NSCs), which were shown to have a natural tropism toward tumor cells and the capability to penetrate the center of the tumor mass [8–11]. In our previous studies, we showed that NSCs loaded with CRAd-S-pk7 could travel toward the tumor cells while simultaneously protecting CRAd-S-pk7 from immune surveillance as well as acting as host cells for CRAd-S-pk7 progeny production [8–12]. Furthermore, NSCs loaded with CRAd-S-pk7 could be delivered into the center of the tumor mass and lyse tumor cells more efficiently than virus administration alone, which accounted for a significant survival benefit in xenograft murine models of GBM [1, 8–12].

Given the fact that the murine model is not permissive to replication of human adenoviruses [13–15], we herein investigated the biodistribution and toxicology of CRAd-S-pk7 after intracerebral administration of the therapeutic viral agent into Syrian Hamsters. This study was conducted under FDA guidelines, using Good Laboratory Practice (GLP) throughout, and will be used in filing of the final Investigational New Drug (IND). This Syrian Hamster model is permissive for human adenovirus replication and is fully immune competent [9, 15], so the replication profiles and immune responses in different organs after the administration of this vehicle can recapitulate the replication kinetics and the toxicity of CRAd-S-pk7 in the human clinical setting. For this analysis, we administered single intracerebral doses of 2.5 × 10^7^, 2.5 × 10^8^, and 2.5 × 10^9^ viral particles (vp) per animal. These doses were established based on our preclinical analysis using 50 vp/cell of CRAd-S-pk7 per 5 × 10^5^ NSCs (2.5 × 10^7^ vp/hamster in total) [9].

In the immunological analysis, antibody response against E1 of the vector was observed in a dose-dependent manner as expected. Pathologically, minimal and transient reactive inflammatory changes were observed at all dose levels, which were not toxicologically significant. Furthermore, the viral vector was predominantly localized to the brain, with low levels of vector DNA observed in other tissues. Presence of vector in tissues was not correlated with any microscopic changes or vector-mediated toxicity. In summary, we demonstrated that the agreeable safety profile of CRAd-S-pk7 justifies proceeding with a planned Phase I clinical trial, utilizing this agent loaded onto FDA-approved NSCs for patients with GBM.

## Methods

### Animals

Syrian hamsters (Haslett, MI, USA) (n = 250) were randomized by body weight into one of four groups (groups 2–5) comprised of 120 male and 120 female hamsters distributed in equal numbers into core and satellite groups (60 male and 60 female hamsters each) (Table 1). The core groups (15 animals/sex/group) were used for toxicology evaluations including hematology and clinical chemistry, and the satellite groups (15 animals/sex/group) were used for assessment of the biodistribution and immunogenicity of the test article (CRAd-S-pk7) as well as the effect on coagulation parameters. Of note, 5 male and 5 female hamsters were arbitrarily placed into Group 1. They remained untreated and were used on day 1 for collection of baseline immunogenicity samples, after which they were removed from the study without further evaluation. All hamsters were approximately 5–6 weeks old on arrival at Southern Research (Birmingham, AL, USA) and the study was approved by the Institutional Animal Care and Use Committee. The hamsters in groups 2–5 were given an intracerebral injection of vehicle [GST150 Buffer (20 mM Tris, 150 mM NaCl, 2.5 % (w/v) glycerol, pH 8.0)] or CRAd-S-pk7 at 2.5 × 10^7^, 2.5 × 10^8^, or 2.5 × 10^9^ viral particles (vp)/animal, respectively, at a fixed volume on day 1. Blood samples were drawn on days 6, 34, or 62 prior to necropsy from 5 male and 5 female hamsters from each of the core groups for the assessment of hematology and clinical chemistry parameters/analytes, and from another five male and five female hamsters in the satellite groups for the assessment of coagulation parameters.

**Table 1:** Assignment of hamsters to study groups

Group	Treatment	CRAd-S-pk7 dose (vp/animal)^a^	Number of animals						
			Core groups			Satellite groups			
			Day 6 necropsy	Day 34 necropsy	Day 62 necropsy	Day 1	Day 6 necropsy	Day 34 necropsy	Day 62 necropsy
1	Untreated	0	–	–	–	5 M/5 F	–	–	–
2	Vehicle	0	5 M/5 F	5 M/5 F	5 M/5 F	–	5 M/5 F	5 M/5 F	5 M/5 F
3	Vector low	2.5 × 10^7^	5 M/5 F	5 M/5 F	5 M/5 F	–	5 M/5 F	5 M/5 F	5 M/5 F
4	Vector mid	2.5 × 10^8^	5 M/5 F	5 M/5 F	5 M/5 F	–	5 M/5 F	5 M/5 F	5 M/5 F
5	Vector high	2.5 × 10^9^	5 M/5 F	5 M/5 F	5 M/5 F	–	5 M/5 F	5 M/5 F	5 M/5 F

Thirty hamsters per sex were assigned to each of four dose groups, and were administered one intracerebral injection on day 1 of either vehicle (group 2) or the test article at 2.5 × 10^7^, 2.5 × 10^8^, or 2.5 × 10^9^ viral particles (vp)/animal (groups 3, 4, and 5, respectively). Of these animals, 15 animals/sex/group (core groups) were used for toxicology evaluations including hematology and clinical chemistry, and the remaining animals (satellite groups) were used for assessment of the biodistribution and immunogenicity of the test article as well as the effect on coagulation parameters. In addition, five untreated animals per sex (group 1) were used on day 1 for collection of baseline immunogenicity samples, after which they were removed from the study without further evaluation

*VP* viral particles, *M* males, *F* females

^a^The low, mid, and high vector doses were selected to bracket the expected human clinical range of 6.8 × 10^10^ to 2.06 × 10^11^ vp/dose, with doses scaled based on the relative brain weights of the two species (hamster ≈ 0.001 × human)

### Clinical observations

All animals were observed at least twice daily during the pre-study and study periods for signs of mortality and moribundity.

### Body weights and food consumption

All animals were weighed on day 1 and randomly assigned to each group. During the test periods, each animal was weighed weekly. Quantitative food consumption was measured weekly beginning on day 1 for each animal in the core group. Values were reported as an average consumption (grams/animal/day).

### Tissue preservation and processing

Following injection of viral particles (2.5 × 10^7^, 2.5 × 10^8^, and 2.5 × 10^9^, respectively), five male and female hamsters per group were sacrificed at 6, 34, and 62 days. Whole brains, as well as other organs, were collected for tissue bio-distribution analysis. Tissues (bone marrow (bilateral femurs; flushed), gonads, heart, incision site (skin, subcutis, muscle), kidneys, liver, lungs, mesenteric lymph nodes, and spleen) were snap frozen in liquid nitrogen and stored at −70 °C until analysis.

### Viral DNA and transcript detection in the brain

Five male and five female animals per group were sacrificed at 6, 34, and 62 days. Whole brains were collected for detection and quantification of viral DNA at each time point. Tissues were snap frozen in liquid nitrogen and stored at −70 °C, or colder, until analysis. Brain tissue was analyzed for viral DNA via quantitative PCR (qPCR), using an assay previously evaluated and validated at Southern Research Institute. Tissue was homogenized and DNA was extracted using the Qiagen QIA amp 96 DNA QIAcube HT Kit (Qiagen, Venlo, The Netherlands). Samples were analyzed in triplicates, with one replicate spiked with 100 copies of adenovirus DNA to evaluate for possible inhibitors. The Qiagen QuantiTect Multiplex Master Mix was used for all qPCR reactions (Qiagen, Venlo, The Netherlands). Primers, probe, and control DNA were added to each reaction to serve as an internal amplification control and to rule out inhibition. DNA concentration was measured by spectrophotometer. The probes and primers used were validated specifically for human adenovirus serotype five hexon gene.

All brains were analyzed on days 6 and 34. Only those positive for vector DNA on day 34 were analyzed on day 62. PCR analysis was conducted using Applied Biosystems 7900HT Fast Real-Time PCR System (Applied Biosystems, Foster City, CA, USA) with software SDS 2.2.2. Settings used were: activation at 95 °C for 15 min, 45 cycles of denaturing at 95 °C for 10 s, and annealing/elongation at 60 °C for 45 s.

### Systemic biodistribution of virally encoded transcripts after CRAd-S-pk7 intracerebral injection

On the planned day of necropsy, five male and five female animals in the satellite groups 2–5 were anesthetized and blood samples (~0.5 mL) were collected from the retro-orbital sinus into anticoagulant tubes (EDTA). Samples were mixed by gentle inversion, then snap frozen on dry ice and stored at −70 °C or below until analysis. The animals were then euthanized and the following whole tissues were collected: bone marrow, femur (both; flushed), brain, gonads (ovary/testis), heart, incision site (skin, subcutis, muscle), kidney, liver, lungs, lymph nodes, mesentery, and spleen. Tissue samples were snap frozen in liquid nitrogen and stored at −70 °C in the same fashion as blood samples. Blood and tissues collected were assayed for the presence of vector DNA using a qPCR protocol previously evaluated and validated at Southern Research Institute. Tissue was homogenized and DNA was extracted using the Qiagen QIA amp 96 DNA QIAcube HT Kit (Qiagen, Venlo, The Netherlands). Samples were analyzed in triplicates, with one replicate spiked with 100 copies of adenovirus DNA to evaluate for possible inhibitors. The Qiagen QuantiTect Multiplex Master Mix was used for all qPCR reactions (Qiagen, Venlo, The Netherlands). Primers, probe and control DNA were added to each reaction to serve as an internal amplification control and to rule out inhibition. DNA concentration was measured by spectrophotometer. The probes and primers used were validated specifically for human adenovirus serotype five hexon gene.

Since brain was the only tissue positive for vector DNA at day 34, blood and other tissue samples were not assayed at day 62. PCR analysis was conducted using Applied Biosystems 7900HT Fast Real-Time PCR System (Applied Biosystems, Foster City, CA) with software SDS 2.2.2. Settings used were: activation at 95 °C for 15 min, 45 cycles of denaturing at 95 °C for 10 s and annealing/elongation at 60 °C for 45 s.

### Blood collection

On days 6, 34, and 62 post-injection, blood samples were collected prior the euthanasia for hematology and clinical chemistry evaluation from each hamster scheduled for necropsy. On these days blood was also collected from each hamster in satellite groups for evaluation of coagulation prior to euthanasia. Blood was collected from each hamster from the retro-orbital sinus using tubes containing either EDTA as an anticoagulant for hematology, or no coagulant for clinical chemistry, or sodium citrate as an anticoagulant for coagulation evaluation. Tubes were mixed by gentle inversion upon collection. Clinical pathology samples were analyzed on the same day that the samples were obtained. The following hematology parameters were assayed: total leukocyte count, erythrocyte count, hemoglobin, hematocrit, mean corpuscular volume, mean corpuscular hemoglobin, mean corpuscular hemoglobin concentration, reticulocyte count, platelet count, differential leukocyte counts, RBC morphology, and nucleated red blood cell count. The following clinical chemistry parameters were assayed: urea nitrogen, aspartate aminotransferase, alanine aminotransferase, alkaline phosphatase, glucose, creatinine, creatine kinase, total protein, albumin, globulin (calculated), albumin/globulin ratio (calculated), sodium, potassium, chloride, cholesterol, total bilirubin, calcium, phosphorus, blood urea nitrogen (BUN)/creatinine ratio (calculated). For coagulation analysis, prothrombin time and fibrinogen were assayed.

### Immunogenicity

Control blood samples for immunogenicity analysis (~0.5 mL) were collected on day 1 from five animals per sex in the untreated group. Terminal blood samples were collected from satellite group animals on days 6, 34, and 62 immediately after blood collection for biodistribution. Samples were collected into tubes without anticoagulant. Serum was frozen and stored at −70 °C until analysis. One aliquot of each sample was assayed for antibodies against the vector using an ELISA assay developed and validated for GLP use at Southern Research.

### Histopathology and toxicity

In the pathologic evaluation of CRAd-S-pk7 when administered as a single intracerebral dose in hamsters, viral toxicity was assessed through both gross and microscopic evaluations. Half of the animals were used for tissue collection for histopathology and the remaining satellite animals were used for biodistribution studies. The doses of vectors administered to groups 2 through 5, respectively, were: 0 (vehicle), 2.5 × 10^7^ (vector, low), 2.5 × 10^8^ (vector, mid), or 2.5 × 10^9^ (vector, high). Animals were then euthanized on days 6, 34, and 62 and examined post-mortem.

For each post-mortem pathologic examination at the respective time points, all tissues were examined both grossly and microscopically. Hematoxylin and eosin (H&E) staining was performed on formalin-fixed paraffin-embedded (FFPE) tissue for histopathological evaluation. In addition to the standard tissue evaluation, target organs (brain and cervical spinal cord) were evaluated on day 34 and any gross lesions were documented, examined, and histologically graded on day 62. Lesions were graded according to their degree of involvement.

Microscopic lesions observed in this study were graded using a numerical scoring system in which 1 = minimal, 2 = mild, 3 = moderate, and 4 = marked. In general, lesions that affected less than 10 % of the tissue were considered as minimal, lesions that affected 11–50 % of tissue were considered as mild, lesions that affected 51–75 % of tissue were considered to as moderate, and lesions that affected greater than 75 % of tissue were considered as marked. All graded pathological evaluation was blinded to treatment.

### Statistical analyses

For CRAd-S-pk7 dose effect analyses, cytotoxicity and clinical pathology data among four groups (groups 2, 3, 4, and 5) were compared. When the equality of variances among the groups assumption holds, One-way analysis of variance (ANOVA) with post hoc Bonferroni correction for multiple comparison were used. When the variances among the groups were not equal, Kruskal–Wallis test with post hoc Mann–Whitney *U* tests with the Bonferroni correction were performed. Linear regression models were conducted to assess the effect of the four groups on viral copies with log_10_ transformed. Generalized Estimating Equation approach was used to estimate the parameters in the regression models taking into account the longitudinal structure of the data. For the hematology, clinical chemistry, and coagulation parameters/analytes, the individual’s percent change (%Δ) on each day of analysis relative to the vehicle group’s (i.e., group 2) mean for the time point by sex was calculated with the following formula: %Δ in parameter/analyte for individual on day X = [(individual’s day X value − Y)/Y] × 100, where X = days 6, 34, or 62 and Y = vehicle group mean value for day X by sex and time point. Statistical analyses were performed with the statistical analysis system (SAS) software Version 9.4 (SAS Institute Inc., Cary, NC, USA).

## Results

### In vivo effects of CRAd-S-pk7 in a permissive immunocompetent animal model

**Fig. 1:**
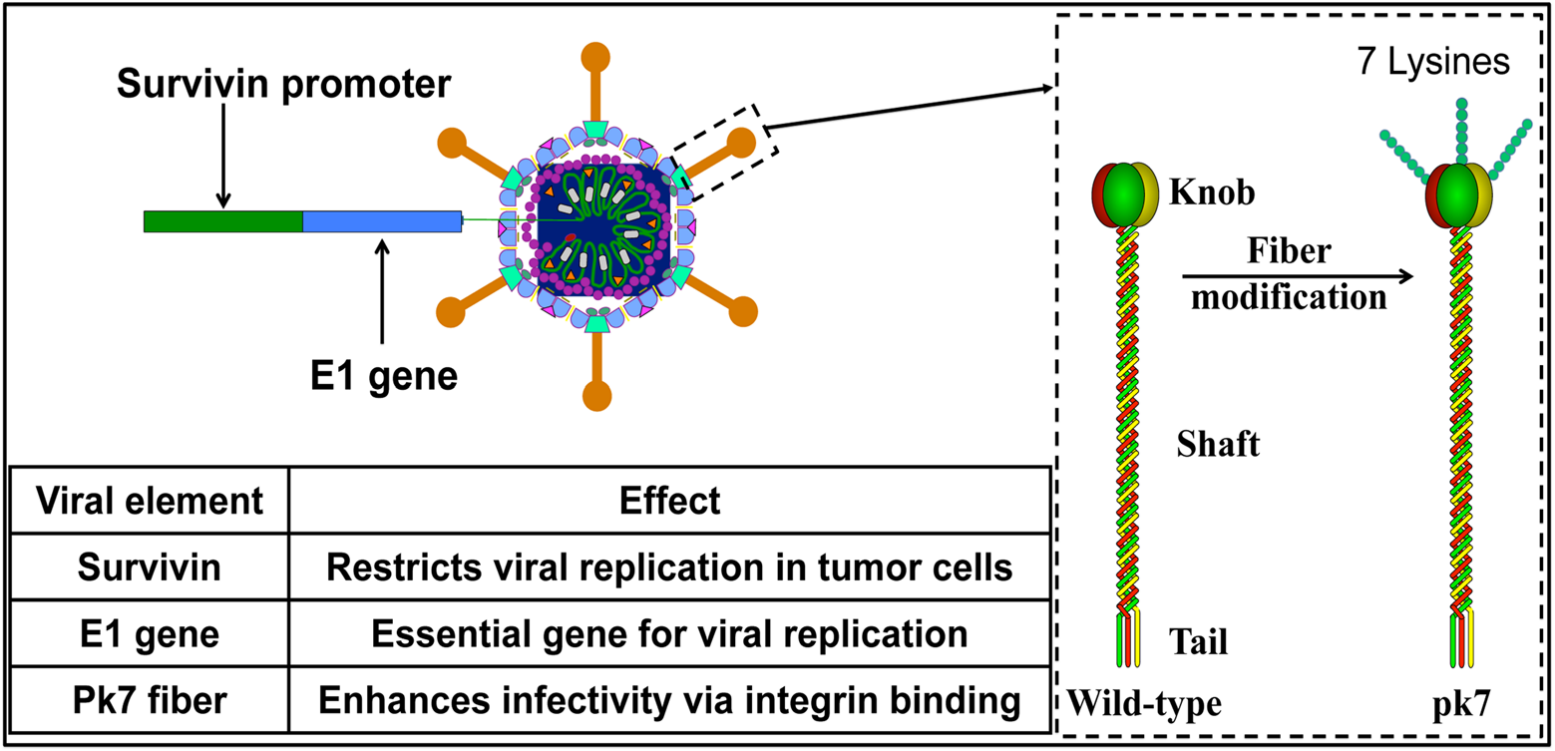
Schematic of genome of CRAd-S-pk7. The fiber knob domain has seven lysine residues added to the C-terminal end to enhance the infectivity of the virus. The essential early replication gene (E1) is under the transcriptional control of the tumor-specific promoter survivin (S) to restrict viral replication to tumor cells (oncolysis)

**Fig. 2:**
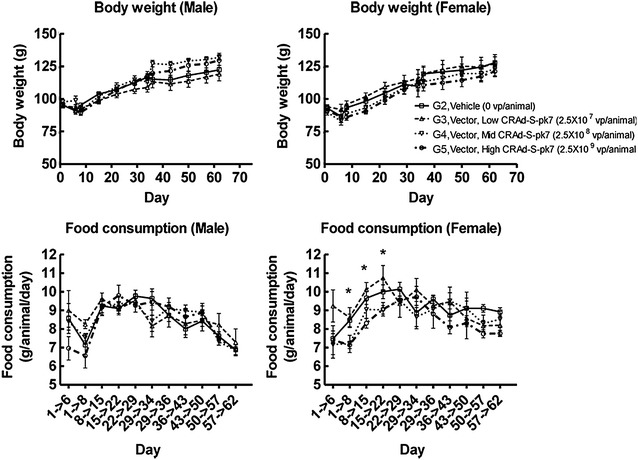
Body weight and food consumption for male and female hamsters. Administration of CRAd-S-pk7 had no significant effect on body weight or food consumption for male hamsters (P > 0.05, one-way ANOVA). In the female group, a significant difference was noted concerning decreased food consumption on days 1–8: groups 2 vs. 4, P 0.032; groups 2 vs. 5, P 0.027; days 8–15: groups 2 vs. 5, P 0.009; days 15–22: groups 3 vs. 5, P 0.01; one-way ANOVA. However, this tendency was not considered to be biologically significant. The analyzed measurements are shown as g for body weight and g/animal/day for food consumption. *Each plot* represents the average of 5–10 animals/sex/group/time point and standard deviations were indicated by *error bars*. One-way ANOVA and Kruskal–Wallis test were used for comparison between vehicle (G2) and treated (G3-5) groups. *P < 0.05

The human adenovirus serotype five was genetically modified to enhance its infectivity and to restrict its replication to glioma as shown in Fig. 1. CRAd-S-pk7 infects cells by binding to anionic cell surface proteins through seven lysine residues (pk7) on the adenoviral fiber and subsequently initiating replication by way of *E1* gene expression under the control of the tumor-specific promoter, survivin [4, 16]. To investigate the overall health changes on test subjects, we administered this therapeutic viral agent intracerebrally to Syrian hamsters, a human adenovirus replication-permissive animal model, in single doses (Table 1) and monitored mortality, clinical signs, food consumption, and body weight. Although a few animals showed scabs at the surgical site, those scabs were considered to be related to the surgical procedure. Therefore, there was no dose-related pattern associated with this observation. Only CRAd-S-pk7-treated female hamster groups showed a tendency to consume less food at most time points than the vehicle control female group did (days 1–8: groups 2 vs. 4, P 0.032; groups 2 vs. 5, P 0.027; days 8–15: groups 2 vs. 5, P 0.009; days 15–22: groups 3 vs. 5, P 0.01; one-way ANOVA, respectively). However, this tendency was not considered to be biologically significant. Also, there was no observable effect on body weights of CRAd-S-pk7-administered hamsters in this study (P > 0.05, one-way ANOVA, Kruskal–Wallis test when appropriate) (Fig. 2).

### Detection of viral DNA in the brain

**Fig. 3:**
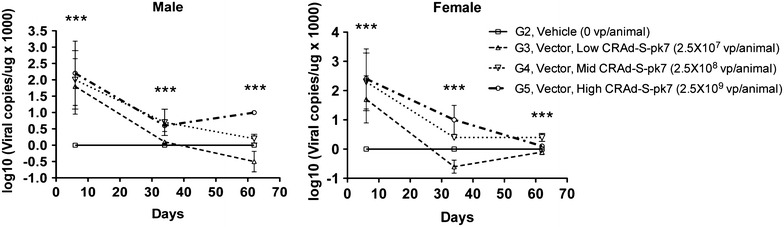
Mean vector DNA in the brains. Brain tissues of male and female hamsters were analyzed for the presence of CRAd-S-pk7 DNA post virus injection. Intracerebral CRAd-S-pk7 DNA was found 6 days after injection in both sexes (G2 vs. G3/4/5, P < 0.0001, one-way ANOVA), whereas DNA detection decreased gradually by days 32 and 64 by at least one order of magnitude (G2 vs. G3/4 for male hamsters, P < 0.0001, one-way ANOVA, and G2 vs. G5 for female hamsters, P < 0.0001, one-way ANOVA). The analyzed measurements are shown as log_10_ (viral copies/ng × 1000). *Each* *plot* represents the average of five animals/sex/group/time point and standard deviations were indicated by error bars. One-way ANOVA and Kruskal–Wallis test were used for comparison between vehicle (G2) and treated (G3-5) groups. ***P < 0.001

Identification and quantitative analysis of viral DNA in the brain was performed to assess the ability of the virus to replicate and persist for the duration of the study period. The viral transcripts present were compared in the brains of control, vehicle-treated, and virus-treated animals via tissue homogenization, DNA extraction, and qPCR as described in the methods section. Viral DNA was not detectable in the brains of control and vehicle-treated animals for the duration of the study. Dose-dependent copies of viral DNA were found in the brains of all animals receiving treatment, with the exception of one in the 2.5 × 10^8^ vp/animal group, at 6 days. All brain tissues remained positive for viral DNA at 34 and 62 days, P < 0.0001, G2 vs. G3/4/5, one-way ANOVA. There was a decrease in viral DNA detected by 1–2 orders of magnitude when comparing days 6 and 34 (10^2−4^ vs. 10^0−1.5^) in both male and female hamsters, G2 vs. G3/4/5, P = 0.0002 and P < 0.0001, one-way ANOVA, respectively. However, a relevant amount of virus was still present on day 34 when comparing vehicle and treatment groups (G2 vs. G3/4/5, P < 0.0001, one-way ANOVA). In addition, there was a significant decline in the viral DNA present from days 34 to 62 in female (10^1.5^ vs. 10^0^, G2 vs. G5, P < 0.0001, one-way ANOVA) and male hamsters (10^1^ vs. 10^0^, G2 vs. G3/4, P < 0.0001, one-way ANOVA) (Fig. 3).

### Systemic biodistribution of virally encoded transcripts after CRAd-S-pk7 intracerebral injection

**Fig. 4:**
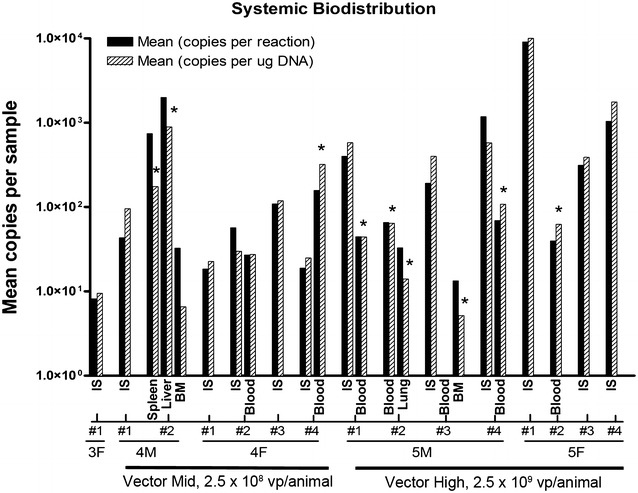
Biodistribution of vector DNA. Quantitative PCR values for mean copies per reaction and mean copies per μg of DNA for systemic organs and tissues at day 6 after vector administration. Tissues listed are those positive for viral DNA in each animal. Animals are delineated by group number and gender. The analyzed measurements are shown as mean copies per sample in the log_10_ scale. *Each plot* represents the average of duplicate samples and standard deviations are indicated by *error bars*. One-way ANOVA and Kruskal–Wallis test were used for comparison between viral copies in different organs/blood and those present at the injection site. *P < 0.05. *G4* Vector mid 2.5 × 10^8^ vp/animal, *G5* vector high 2.5 × 10^9^ vp/animal, *IS* injection site, *BM* bone marrow

Evaluation for viral DNA in the blood and systemic tissues was performed to determine viral distribution, replication, and potential toxicity in off-target tissues. All samples from animals treated with the control vehicle remained negative for vector DNA throughout the study. In the satellite groups receiving test article, vector DNA was detected in various peripheral tissues on day 6 (Fig. 4). It was detected in the liver (9 × 10^2^ copies/μg of DNA, P < 0.05 vs. IS 10^0^ copies/μg of DNA, one-way ANOVA), spleen (3 × 10^2^ copies/μg of DNA, P < 0.05 vs. IS), and bone marrow (9 × 10^0^ copies/μg of DNA, P > 0.05 vs. IS) in one male from the 2.5 × 10^8^ vp/animal dose group. This animal had no detectable vector DNA in the brain or other tissues. Analysis of additional animals revealed two females in the 2.5 × 10^8^ vp/animal dose group (6 × 10^1^ viral copies/μg of DNA in the blood vs. 6.3 × 10^1^ at IS, P > 0.05; 6 × 10^2^ copies/μg of DNA in the blood vs. 4 × 10^1^ at IS, P < 0.05, one-way ANOVA) and three males (7 × 10^1^ viral copies/μg of DNA in the blood vs. 9 × 10^2^ at IS, P < 0.05; 9 × 10^1^ copies/μg of DNA in the blood vs. 10^0^ at IS, P < 0.05, 2 × 10^2^ copies/μg of DNA in the blood vs. 8 × 10^2^ at IS, P < 0.05, one-way ANOVA) and one female in the 2.5 × 10^9^ vp/animal dose group (9 × 10^1^ viral copies/μg of DNA in the blood vs. 10^0^ at IS, P < 0.05) that had viral vectors detected in the blood. One male in the 2.5 × 10^9^ vp/animal dose group had viral DNA detected in the lungs (2.7 × 10^1^ viral copies/μg of DNA in the lung vs. 10^0^ at IS, P < 0.05); and one male in the 2.5 × 10^9^ vp/animal dose group had viral vectors present in the bone marrow (8 × 10^0^ viral copies/μg of DNA in the BM vs. 8 × 10^2^ at IS, P < 0.05, one-way ANOVA). On day 34, only brain samples were positive for vector DNA. The day 6 results indicate that the vector was able to enter the systemic circulation in a small number of animals. In these cases, viral particles were found largely in expected organs, those with robust vascular supply and known depot or scavenger function. Further, the presence of vector was not correlated with any microscopic changes in the tissues that would suggest toxicity after CRAd-S-pk7 intracerebral injection. The absence of microscopic changes in these organs confirms the specificity of the vector for glioma, with no sequelae of infection in other tissues.

### Hematological and clinical chemistry analyses of CRAd-S-pk7 treatment

**Fig. 5:**
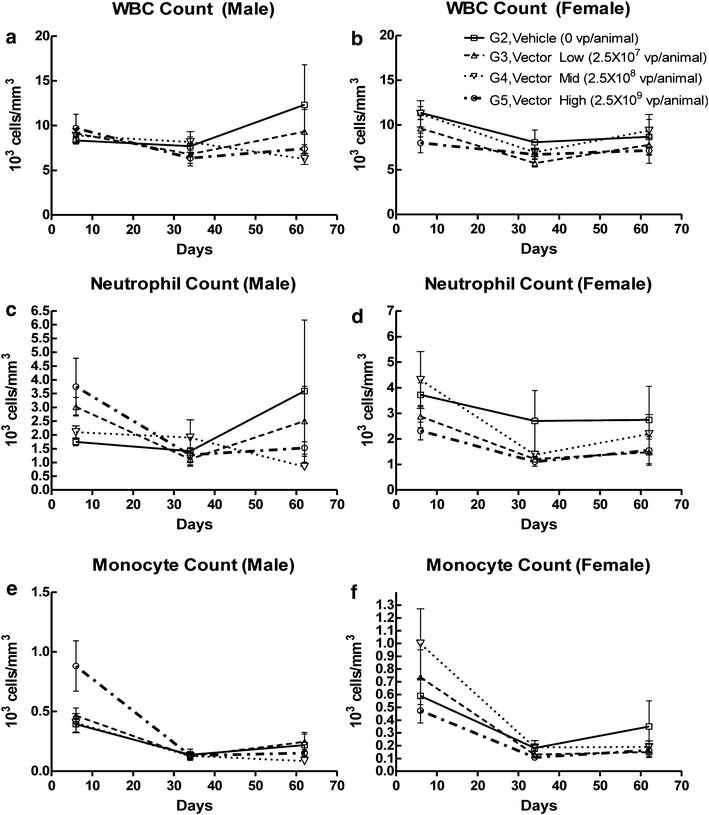
Evaluation of inflammatory response to CRAd-S-pk7 administration over time. Cell counts for total white blood cells (WBC) (**a**, **b**) neutrophils (**c**, **d**), and monocytes (**e**, **f**) were determined from the peripheral blood of both male and female hamsters in vehicle and vector-treated groups on days 6, 34, and 62 for the doses shown (n = 5 animals/group). The analyzed measurements are shown as number of cells per mm^3^ (×10^3^). Standard deviations are indicated by *error bars*. One-way ANOVA and Kruskal–Wallis test were used for comparison of cell counts between animals from vehicle (G2) and treated (G3-5) groups; ns, P > 0.05

To determine whether administration of CRAd-S-pk7 elicited an inflammatory response, WBC, neutrophil, monocyte counts, as well as coagulation and other hematology parameters were obtained from peripheral blood samples. Clinical chemistry parameters were also assessed in order to evaluate potential adverse effects of CRAd-S-pk7 treatment. No significant differences in WBC, neutrophil, monocyte counts, or other hematological parameters were observed between vehicle and treatment groups for most groups, P > 0.05, one-way ANOVA. Although a slight increase in monocyte counts was observed in male hamsters from group 5 and female hamsters from group 3 on day 6 after treatment, the difference was not statistically significant as compared to the vehicle control group (G2) (P = 0.15, one-way ANOVA). In addition, such an increase in monocyte counts was not detected at later time points. Although elevated WBC, neutrophil, and monocyte counts relative to published reference levels were observed in some animals, there was no clear implication that CRAd-S-pk7 administration was related to the increased cell counts. These results suggest that there is no significant inflammatory response to the administration of CRAd-S-pk7. CRAd-S-pk7 also does not affect coagulation parameters, as no significant changes in fibrinogen levels or prothrombin were observed at any time point in hamsters that were administered with any dose of CRAd-S-pk7 compared to control (P > 0.05, one-way ANOVA) (Fig. 5a–f).

Likewise, in almost all clinical chemistry parameters measured (urea nitrogen, aspartate aminotransferase, alanine aminotransferase, alkaline phosphatase, glucose, creatinine, creatine kinase, total protein, albumin, globulin (calculated), albumin/globulin ratio (calculated), sodium, potassium, chloride, cholesterol, total bilirubin, calcium, phosphorus, blood urea nitrogen (BUN)/creatinine ratio (calculated)) at any time point, there was no observable difference between any of the groups (P > 0.05, one-way ANOVA). In addition, serum albumin was significantly decreased in male hamsters administered the highest vector dose (2.5 × 10^9^ vp/animal, G5) at day 6 (3.22 ± 0.13 in G5 vs. 3.02 ± 0.08 in G2, P < 0.05, one-way ANOVA). However, the effect size was small and not considered to be of biological importance (data no shown).

### Induction of a moderate IgG antibody response with CRAd-S-pk7 treatment

**Fig. 6:**
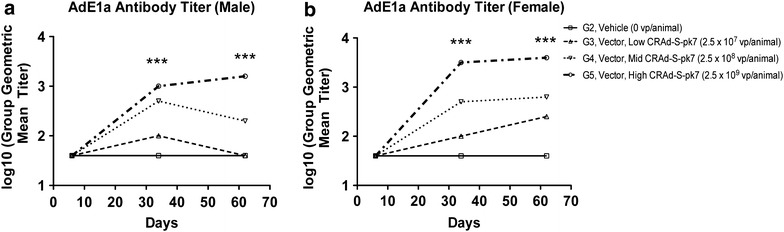
Moderate dose-dependent antibody response to CRAd-S-pk7 administration. Serum anti-AdE1a antibody titer was determined in both male and female hamsters (**a**, **b**) on days 6, 34, and 62 after initial treatment for the doses shown (n = 5 animals/group). The analyzed measurements are shown as group geometric mean titers in the log_10_ scale. One-way ANOVA and Kruskal–Wallis test were used for comparison of E1A antibody titers between animals from vehicle control (G2) and treatment (G3-5) groups; ***P < 0.001. Values of the group geometric mean titer were rounded to the nearest whole number. For the calculation of the geometric mean, when the titer was <400 (the lower limit of detection in this assay), a value of 40 (1/10th of 400) was used

We next sought to investigate the immunogenicity of CRAd-S-pk7 by determining serum antibody responses to Ad-E1a in control and treatment hamsters across all three time points. No Ad-E1a antibody response was observed in untreated hamsters or vehicle-treated/G2 hamsters. Furthermore, no Ad-E1a antibody response was observed in hamsters treated with any dose level of CRAd-S-pk7 at day 6, P > 0.05, one-way ANOVA. Animals treated with 2.5 × 10^8^ and 2.5 × 10^9^ vp/animal (the middle/G4 and high/G5 vector doses) exhibited increased anti-AdE1a antibody titers in a dose-dependent manner on days 34 and 62 in both males (on day 34: 1.6 for G2 vs. 2.7 for G4, P < 0.0001; and 1.6 for G2 vs. 3 for G5, P < 0.0001; on day 62: 1.6 for G2 vs. 2.3 for G4, P = 0.0062; and 1.6 for G2 vs. 3.2 for G5, P < 0.0001, one-way ANOVA) and females (on day 34: 1.6 for G2 vs. 2.7 for G4, P = 0.002; and 1.6 for G2 vs. 3.5 for G5, P < 0.0001; on day 62: 1.6 for G2 vs. 2.8 for G4, P = 0.004; and 1.6 for G2 vs. 3.6 for G5, P < 0.0001, one-way ANOVA). In animals treated with the low vector dose of 2.5 × 10^7^ vp/animal, viral titers were close to background levels on both days 34 and 62, P < 0.05. These data suggest that intracerebral injection of CRAd-S-pk7 vector results in a marginal IgG immune response for the vector at low doses and a stronger IgG immune response for the middle and high dose groups (Fig. 6).

### Gross and microscopic histopathological analyses

**Fig. 7:**
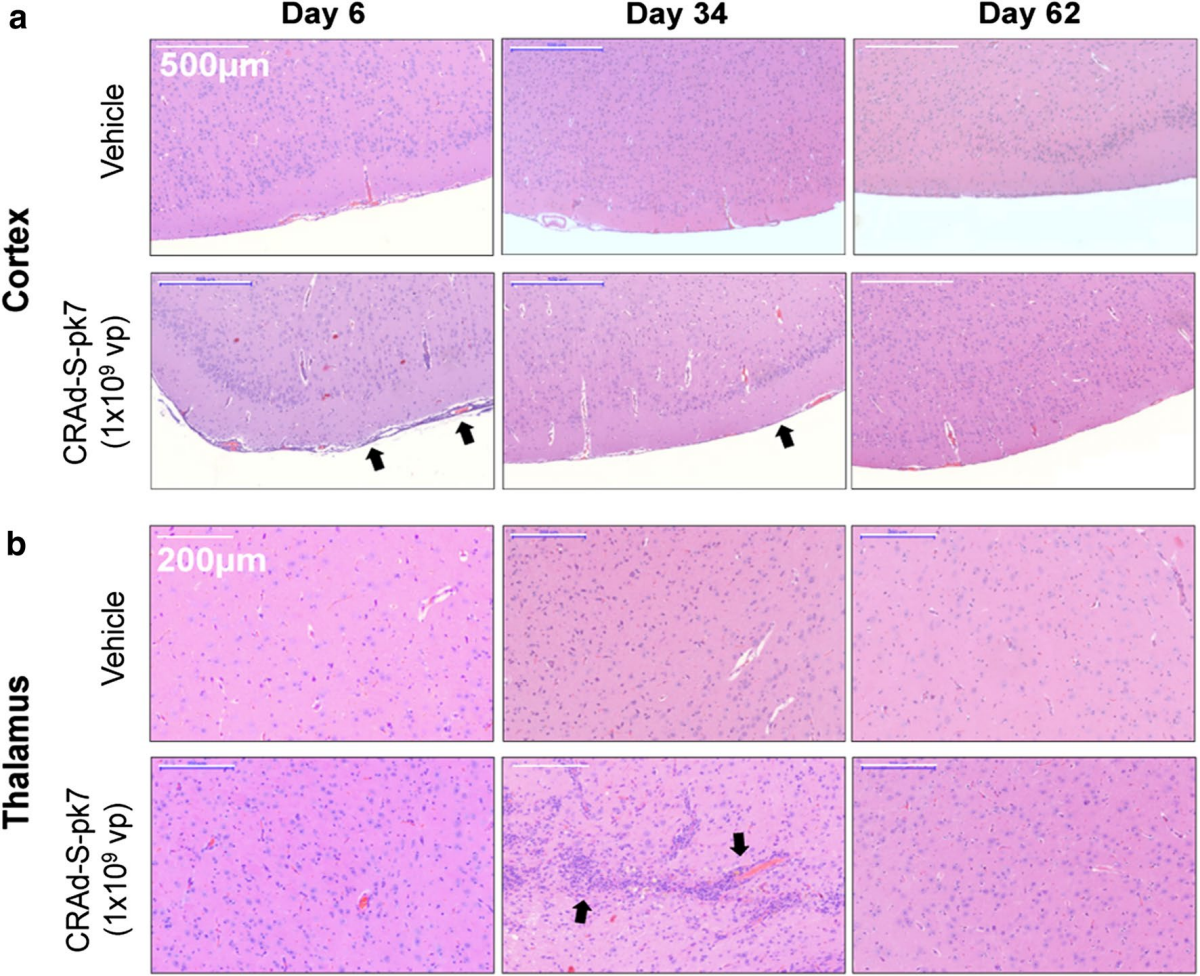
Histological analysis of samples associated with CRAd-S-pk7 adenoviral vector delivery over time. Assessment of histopathological changes on CNS tissues associated with adenoviral vector treatment. Vector high dose group (1 × 10^9^) is shown. In **a** minor amounts of meningitis perivascular inflammation were detected in the treatment groups, which subsided by 62 days post treatment. In **b** minor inflammation of the thalamus and its associated vasculature occurred in select animals; which also subsided by 62 days post treatment. Quantification of events is outlined in Additional file 1: Table S2. For histological examination, we used 10 hamsters per group per time point

To determine if viral vector treatment caused any significant pathology, hamsters were sacrificed at predetermined time points (days 6, 34, and 62), and all organs were first observed macroscopically before fixation/sectioning/H&E staining for microscopic analysis. Standard gross pathologic evaluation revealed no associated pathogenesis as a result of viral vector toxicity (Additional file 1: Table S1). There was focal inflammation at the injection sites of all treatment groups, including the vehicle cohort, which suggests that most of the gross inflammatory effects were due to the mechanical trauma of the procedure rather than the therapeutic agent (Additional file 1: Table S2). Microscopically, there were treatment-related effects observed in some animals. On day 6, there was chronic perivascular and meningeal inflammation, including a cytopathic effect consisting of eosinophilic intranuclear inclusions, consistent with adenoviral infection in the brain. Both acute and chronic inflammation was noted along with histiocytic inflammatory infiltrates. Mild to moderate chronic inflammation was observed in the meninges along with mild to moderate chronic perivascular inflammation in the brains of most animals of all test article groups (Additional file 1: Table S2). Minimal chronic perivascular inflammation was observed in the cervical, thoracic, and lumbar spinal cords (and meninges) of select high vector animals (data not shown). Mild perivascular inflammation was also observed in the pituitary glands (pars nervosa) of such high vector animals. The occurrence of cells with large, smudged nuclei and atypical nuclear contours along with occasional intranuclear inclusions was also observed in the brains of select animals. Microscopic evaluation on day 34 revealed similar or lessened chronic inflammatory patterns that were further reduced by Day 62 (Additional file 1: Table S2; Fig. 7).

### Analysis of lethality and toxicity towards organs and tissues

To demonstrate that CRAd-S-pk7 did not result in animal death and/or organ-specific toxicity, a study cohort set of 30 hamsters of each sex per group (120 males and 120 females) was evaluated with regard to clinical signs and symptoms associated with the treatment. All hamsters were sacrificed at their pre-determined endpoints with no clinically relevant manifestations (Table 2). Also, hematological studies and clinical chemistry work-up did not show evidence of viral toxicity either. Anatomic pathological evaluation revealed reactive regenerative morphologic changes resultant from mechanical trauma due to the intracerebral injection of the viral vector and cytopathic effects consistent with cellular adenoviral infection, although no diagnostic anomalies associated with vector toxicity were observed. No morbidity or mortality was experienced in animals prior to their sacrificial endpoints, demonstrating that no lethality resulted from the test article. This is consistent with the anatomic and pathological studies that demonstrated no identifiable vector-mediated toxicity in any animals of all test groups.

**Table 2:** Survival of animals treated with CRAd-S-pk7 viral vector after intracranial injection

Days post treatment	Vehicle	1 × 10^7^ VP	1 × 10^8^ VP	1 × 10^9^ VP	Survival (%)
Day 6	10/10	10/10	10/10	10/10	100
Day 34	10/10	10/10	10/10	10/10	100
Day 62	10/10	10/10	10/10	10/10	100

Regardless of titer of vector treatment, all animals made it to their predetermined time points (days 6, 34, and 62) without any observable toxicity

*VP* viral particles

## Discussion

We have previously demonstrated the effectiveness of CRAd-S-pk7 as a single or combined therapy against high-grade gliomas [4, 11, 17–19]. As compared with other oncolytic viruses, CRAd-S-pk7 showed the highest level of viral replication and tumor oncolysis in GBM cell lines, with minimal toxicity to normal brain tissue [4, 11, 18]. Intracranial injection of CRAd-S-pk7 alone reduced tumor growth by 300 and 67 % of treated animals were long-term survivors [4, 11, 18]. Synergistic effect of CRAd-S-pk7 with the current standard of care for anti-glioma therapy, radiotherapy and temozolomide, was also positively validated in animal models [17, 19]. Taken together, these findings provide the rationale for further application of this tumor-specific oncolytic virus in a phase 1 clinical trial in patients with recurrent high-grade glioma. In preparation for this, herein we evaluated the biodistribution, toxicology, and anti-viral immune response of CRAd-S-pk7 in Syrian Hamsters, an immunocompetent model that is permissive for the replication of human adenoviruses [11]. We observed that following intracranial administration of vehicle or low, mid, or high doses of the test article formulation of CRAd-S-pk7 (2.5 × 10^7^, 2.5 × 10^8^, or 2.5 × 10^9^ viral particles/animal, respectively), viral particles were largely confined to the brain. Low levels of vector DNA were detected in other tissues in a few animals suggesting that systemic circulation of the virus can occur. Microscopic changes and virus-related toxicity were considered minor and CRAd-S-pk7 intracerebral injection was not associated with lethality. In addition, CRAd-S-pk7 was able to elicit measurable dose-dependent IgG immune response by day 34 after intracranial injection, which was considered to be marginal in the lowest dose group. In sum, the above results provide safety and toxicology data justifying the clinical application of CRAd-S-pk7 in humans with recurrent malignant glioma.

Concerning virus biodistribution, 6 days after intracranial administration, viral genomic DNA was present in the brains of all animals at all time-points, with the exception of one animal. The levels of vector DNA in the brains were dose-dependent and decreased over time, with a maximum decrease observed between days 6 and 34 after intracranial injection. The persistence of virus DNA 62 days after local administration was consistent with low levels of inflammation observed at the viral distribution sites. The significant time-dependent decrease of vector DNA in the brains of the studied animals was consistent with previous reports showing that these conditionally replicative adenovirus vectors are tumor-specific and do not replicate in non-malignant brain tissues [3, 4, 9, 11, 18, 20, 21]. Systemic dissemination of intracranially administered CRAd-S-Pk7 was assessed in immunocompetent hamsters by qPCR. Blood was collected and various organs were harvested from animals sacrificed 6, 34, and 62 days after virus injection. On day 6, vector DNA was present at low levels at the incision site of many animals as well as in the blood and other tissues of a few animals in the mid and high-dose groups. The presence of vector DNA in these non-brain/incision site tissues indicated that for a few animals the vector was able to enter the systemic circulation. However, there were no microscopic lesions in these tissues from core group animals on day 6, suggesting that the presence of vector DNA in extraneural tissues was of no toxicological significance. Moreover, vector DNA was not detected in tissues outside the brain after day 6. These results are consistent with previously published data and confirm the safety and limited biodistribution of CRAd-S-pk7 after intracranial administration [11].

The toxicological profile of CRAd-S-pk7 after intracranial administration was assessed via local and peripheral inflammatory response, measured mainly by immune cell count, fibrinogen and albumin levels, and macro- and microscopic pathological changes in harvested tissues at the above described time points. At all dose levels there was an increase in total leukocyte, neutrophil, and/or monocyte counts and fibrinogen levels, and a significant decrease in mean albumin level for male hamsters in the high dose group on day 6. We also observed microscopic inflammatory lesions in the brain, spinal cord, and meninges for hamsters of both sexes at all three time points (days 6, 34, and 62). However, these observed changes in clinical pathology parameters were for the most part present at relatively low levels. Moreover, there were no clinically significant test article-related inter-group differences for male hamsters on days 34 or 62, suggesting recovery from the adverse effects seen on day 6. There were no apparent test article-related inter-group differences for female hamsters at any time point. Therefore, the observed changes in clinical pathology parameters were considered to be of little toxicological significance other than as possible indicators of the underlying inflammation occurring in the nervous system. Considering additional toxicological parameters, we did not observe any changes in body weight between the studied groups. Changes in hematology, clinical chemistry, and coagulation parameters were minor and transient, and were consistent with the inflammatory changes that were observed microscopically. These changes were considered of little toxicological significance.

In terms of immune reactions, CRAd-S-pk7 was able to elicit a measurable IgG immune response at all dose levels by day 34. The magnitude of this response was related to the dose, with the response in the low dose group considered to be marginal. Because the levels of anti-CRAd-S-pk7 antibodies were higher in the high dose group than in the mid dose on both days 34 and 62, it was not possible from the data in this study to say whether a maximal response had been reached at the high dose. Based on previous reports and the above-described data, it is expected that CRAd-S-pk7 may induce anticancer immunity through viral replication followed by tumor cell lysis in glioma-bearing subjects [22–24]. However, modulating the immune response can be tricky and a fine balance between antiviral and antitumor immune responses must be achieved so that the virus will be able to first infect and lyse the tumor cells and then induce an antitumor immune response against neighboring malignant tissues.

In summary, administration of CRAd-S-pk7 at different doses produced a positive antibody response against the virus. The vector remained localized primarily in the brain and to some degree in the tissues at the incision site, although the presence of low levels of virus DNA in other tissues indicated that the vector was able to enter the systemic circulation in a few animals. However, the presence of virus in those tissues from the satellite group animals was not correlated with any microscopic changes in the same tissues of core group animals that would suggest toxicity of the vector. Test article-related microscopic changes were observed that were consistent with viral disease affecting the central nervous system; these changes appeared to decrease in incidence and severity over time, indicating that recovery was in progress. Because of the microscopic changes seen in the 2.5 × 10^7^ vp/animal group, a no observed adverse effect level (NOAEL) could not be identified for the CRAd-S-pk7 vector under the conditions of this study. These results provide feasibility data related to the biodistribution, toxicology, and immune response of CRAd-S-pk7 in preparation for a phase 1 clinical trial.

## Conclusion

This study provides safety and toxicology data justifying a clinical trial of CRAd-S-pk7 loaded in FDA-approved HB1.F3.CD neural stem cell carriers administered at the tumor resection bed in humans with recurrent malignant glioma.

## Additional file

10.1186/s12967-016-0892-y Hamsters treated with vehicle or differing amounts of CRAd-S-pk7 were observed for any gross pathological observations at the predetermined endpoints (Days 6, 34, and 62). Minimal gross pathological incidents occurred in any of the target organs (CNS). Non-target related pathological changes (testes and uterus) were also seen in the vehicle control groups, suggesting that these observations were unrelated to test article treatment. *N* = 10 hamsters per group/per time point (*n* = 5 male + *n* = 5 female) except in the sex organ groups (*n* = 5 male or *n* = 5 female per time point). In bold are isolated observations potentially influenced by CRAd-S-pk7 treatment. **Table S2.** Hamsters were treated with CRAd-S-pk7 adenoviral vector delivery and were sacrificed at 6, 34, and 62 days post viral vector injection for microscopic pathology observation. Analysis of adverse events associated with vector treatment in the target organs (CNS) is shown above; other organs were analyzed but not included due to lack of any significant viral vector-associated pathology. Analysis shows mild perivascular/meningeal inflammation and mild gliosis in the thalamus/cortex (in bold and shown in Fig. 7), which is largely resolved by day 62 after treatment. *N* = 10 hamsters per group/per time point (*n* = 5 male + *n* = 5 female).
