# Supplementary material for: Single dose GLP toxicity and biodistribution study of a conditionally replicative adenovirus vector, CRAd-S-pk7, administered by intracerebral injection to Syrian hamsters
Source: J Transl Med. 2016 May 16;14:134. doi: 10.1186/s12967-016-0895-8 (PMC4868110; doi:10.1186/s12967-016-0895-8)
Supplement: Supplementary file 1 — 10.1186/s12967-016-0892-y Hamsters treated with vehicle or differing amounts of CRAd-S-pk7 were observed for any gross pathological observations at the predetermined endpoints (Days 6, 34, and 62). Minimal gross pathological incidents occurred in any of the target organs (CNS). Non-target related pathological changes (testes and uterus) were also seen in the vehicle control groups, suggesting that these observations were unrelated to test article treatment. N = 10 hamsters per group/per time point (n = 5 male + n = 5 female) except in the sex organ groups (n = 5 male or n = 5 female per time point). In bold are isolated observations potentially influenced by CRAd-S-pk7 treatment. Table S2. Hamsters were treated with CRAd-S-pk7 adenoviral vector delivery and were sacrificed at 6, 34, and 62 days post viral vector injection for microscopic pathology observation. Analysis of adverse events associated with vector treatment in the target organs (CNS) is shown above; other organs were analyzed but not included due to lack of any significant viral vector-associated pathology. Analysis shows mild perivascular/meningeal inflammation and mild gliosis in the thalamus/cortex (in bold and shown in Fig. 7), which is largely resolved by day 62 after treatment. N = 10 hamsters per group/per time point (n = 5 male + n = 5 female). [file 12967_2016_895_MOESM1_ESM.docx]

| Table S1. **Gross macroscopic observations of target organs and tissues with pathological observations present.** | | | | | |
| --- | --- | --- | --- | --- | --- |
| **Organ** | **Vehicle** | **1x10^7^ VP** | **1x10^8^ VP** | **1x10^9^ VP** | **Days post Tx** |
| Brain | 0/10 | 0/10 | 0/10 | **1/10** | Day 6 |
|  | 0/10 | 0/10 | 0/10 | 0/10 | Day 34 |
|  | 0/10 | 0/10 | 0/10 | 0/10 | Day 62 |
| Spinal Cord Cervical | 0/10 | 0/10 | 0/10 | 0/10 | Day 6 |
|  | 0/10 | 0/10 | 0/10 | 0/10 | Day 34 |
|  | 0/10 | 0/10 | 0/10 | 0/10 | Day 62 |
| Spinal Cord Thoracic | 0/10 | 0/10 | 0/10 | 0/10 | Day 6 |
|  | 0/10 | 0/10 | 0/10 | 0/10 | Day 34 |
|  | 0/10 | 0/10 | 0/10 | 0/10 | Day 62 |
| Spinal Cord Lumbar | 0/10 | 0/10 | 0/10 | 0/10 | Day 6 |
|  | 0/10 | 0/10 | 0/10 | 0/10 | Day 34 |
|  | 0/10 | 0/10 | 0/10 | 0/10 | Day 62 |
| Incision Site | 0/10 | 0/10 | 0/10 | 0/10 | Day 6 |
|  | 0/10 | 0/10 | 0/10 | 0/10 | Day 34 |
|  | 0/10 | 0/10 | 0/10 | 0/10 | Day 62 |
| Testes/Epididymis | 1/5 | 1/5 | 2/5 | 1/5 | Day 6 |
|  | 1/5 | 1/5 | 2/5 | 2/5 | Day 34 |
|  | 2/5 | 0/5 | 1/5 | 1/5 | Day 62 |
| Uterus with Cervix | 3/5 | 3/5 | 1/5 | 3/5 | Day 6 |
|  | 0/5 | 0/5 | 1/5 | 0/5 | Day 34 |
|  | 0/5 | 1/5 | 0/5 | 1/5 | Day 62 |
| Eyes + Optic Nerve | 0/10 | 0/10 | 0/10 | 0/10 | Day 6 |
|  | 0/10 | 0/10 | 0/10 | 0/10 | Day 34 |
|  | 0/10 | 0/10 | 0/10 | 0/10 | Day 62 |
| Liver | 0/10 | 0/10 | **1/10** | 0/10 | Day 6 |
|  | 0/10 | 0/10 | 0/10 | 0/10 | Day 34 |
|  | 0/10 | 0/10 | 0/10 | 0/10 | Day 62 |
| Adrenal Gland | 1/10 | 0/10 | 0/10 | 0/10 | Day 6 |
|  | 0/10 | 0/10 | 0/10 | 0/10 | Day 34 |
|  | 1/10 | 0/10 | 0/10 | 0/10 | Day 62 |
| Seminal Vesicle | 0/10 | 0/10 | 0/10 | 0/10 | Day 6 |
|  | 0/10 | 0/10 | 1/5 | 1/5 | Day 34 |
|  | 0/10 | 0/10 | 0/10 | 0/10 | Day 62 |
| Skin | 0/10 | 0/10 | 0/10 | 0/10 | Day 6 |
|  | 1/10 | 0/10 | 0/10 | 0/10 | Day 34 |
|  | 0/10 | 0/10 | 0/10 | 0/10 | Day 62 |
| Lymph Node | 0/10 | 0/10 | 0/10 | 0/10 | Day 6 |
|  | 0/10 | 0/10 | 0/10 | 0/10 | Day 34 |
|  | 0/10 | **1/10** | 0/10 | 0/10 | Day 62 |
| Cranial Deformity | 0/10 | 0/10 | 0/10 | 0/10 | Day 6 |
|  | 0/10 | 0/10 | 0/10 | 0/10 | Day 34 |
|  | 0/10 | 0/10 | 0/10 | **2/10** | Day 62 |

**Legend:** Hamsters treated with vehicle or differing amounts of CRAd-S-pk7 were observed for any gross pathological observations at the predetermined endpoints (Days 6, 34, and 62). Minimal gross pathological incidents occurred in any of the target organs (CNS). Non-target related pathological changes (testes and uterus) were also seen in the vehicle control groups, suggesting that these observations were unrelated to test article treatment. N=10 hamsters per group/per time point (n=5 male + n=5 female) except in the sex organ groups (n=5 male or n=5 female per time point). In bold are isolated observations potentially influenced by CRAd-S-pk7 treatment.

| Table S2. **Microscopic observations of target organs and tissues with pathological observations present.** | | | | | |
| --- | --- | --- | --- | --- | --- |
| **Organ** | **Vehicle** | **1x10^7^ VP** | **1x10^8^ VP** | **1x10^9^ VP** | **Days post Tx** |
| Within Normal Limits | 3/10 | 1/10 | 0/10 | 0/10 | Day 6 |
|  | 6/10 | 0/10 | 1/10 | 0/10 | Day 34 |
|  | 4/10 | 1/10 | 2/10 | 0/10 | Day 62 |
| **Gliosis:**  **Thalamus** | **0/10** | **3/10** | **2/10** | **3/10** | **Day 6** |
|  | **0/10** | **1/10** | **2/10** | **5/10** | **Day 34** |
|  | **0/10** | **3/10** | **3/10** | **4/10** | **Day 62** |
| **Gliosis:**  **Cortex** | **4/10** | **3/10** | **3/10** | **3/10** | **Day 6** |
|  | **0/10** | **2/10** | **1/10** | **2/10** | **Day 34** |
|  | **1/10** | **2/10** | **3/10** | **1/10** | **Day 62** |
| Gliosis:  Caudate | 0/10 | 0/10 | 2/10 | 2/10 | Day 6 |
|  | 0/10 | 1/10 | 1/10 | 1/10 | Day 34 |
|  | 0/10 | 0/10 | 1/10 | 0/10 | Day 62 |
| Gliosis:  Hippocampus | 1/10 | 0/10 | 0/10 | 0/10 | Day 6 |
|  | 1/10 | 0/10 | 0/10 | 0/10 | Day 34 |
|  | 0/10 | 1/10 | 2/10 | 1/10 | Day 62 |
| Hemorrhage:  Meninges | 0/10 | 0/10 | 0/10 | 1/10 | Day 6 |
|  | 0/10 | 3/10 | 0/10 | 2/10 | Day 34 |
|  | 0/10 | 2/10 | 0/10 | 1/10 | Day 62 |
| Hemorrhage:  Thalamus | 0/10 | 1/10 | 1/10 | 2/10 | Day 6 |
|  | 0/10 | 0/10 | 0/10 | 0/10 | Day 34 |
|  | 0/10 | 0/10 | 0/10 | 0/10 | Day 62 |
| Hemorrhage:  Cortex | 2/10 | 1/10 | 1/10 | 1/10 | Day 6 |
|  | 0/10 | 0/10 | 0/10 | 0/10 | Day 34 |
|  | 0/10 | 1/10 | 0/10 | 1/10 | Day 62 |
| Hemorrhage:  Caudate | 0/10 | 0/10 | 0/10 | 2/10 | Day 6 |
|  | 0/10 | 0/10 | 0/10 | 0/10 | Day 34 |
|  | 0/10 | 0/10 | 0/10 | 0/10 | Day 62 |
| Hemorrhage:  Hippocampus | 1/10 | 0/10 | 0/10 | 0/10 | Day 6 |
|  | 0/10 | 0/10 | 0/10 | 0/10 | Day 34 |
|  | 0/10 | 0/10 | 0/10 | 0/10 | Day 62 |
| Acute  Inflammation:  Caudate | 0/10 | 0/10 | 0/10 | 0/10 | Day 6 |
|  | 0/10 | 0/10 | 0/10 | 0/10 | Day 34 |
|  | 0/10 | 0/10 | 0/10 | 0/10 | Day 62 |
| **Chronic**  **Inflammation:**  **Meninges** | **0/10** | **1/10** | **7/10** | **9/10** | **Day 6** |
|  | **0/10** | **5/10** | **6/10** | **10/10** | **Day 34** |
|  | **0/10** | **1/10** | **1/10** | **2/10** | **Day 62** |
| **Chronic**  **Inflammation:**  **Perivascular** | **0/10** | **5/10** | **8/10** | **10/10** | **Day 6** |
|  | **0/10** | **3/10** | **8/10** | **7/10** | **Day 34** |
|  | **0/10** | **1/10** | **5/10** | **4/10** | **Day 62** |
| Chronic  Inflammation:  Choroid Plexus | 0/10 | 0/10 | 1/10 | 1/10 | Day 6 |
|  | 0/10 | 0/10 | 1/10 | 0/10 | Day 34 |
|  | 0/10 | 0/10 | 0/10 | 2/10 | Day 62 |
| Chronic  Inflammation:  Cortex | 0/10 | 0/10 | 0/10 | 1/10 | Day 6 |
|  | 0/10 | 0/10 | 0/10 | 0/10 | Day 34 |
|  | 0/10 | 0/10 | 0/10 | 0/10 | Day 62 |
| Chronic-Active  Inflammation:  Choroid Plexus | 0/10 | 0/10 | 0/10 | 1/10 | Day 6 |
|  | 0/10 | 0/10 | 0/10 | 0/10 | Day 34 |
|  | 0/10 | 0/10 | 0/10 | 0/10 | Day 62 |
| Chronic-Active  Inflammation:  Thalamus | 0/10 | 0/10 | 0/10 | 1/10 | Day 6 |
|  | 0/10 | 0/10 | 0/10 | 0/10 | Day 34 |
|  | 0/10 | 0/10 | 0/10 | 0/10 | Day 62 |
| Chronic-Active  Inflammation:  Cortex | 1/10 | 1/10 | 1/10 | 1/10 | Day 6 |
|  | 0/10 | 0/10 | 0/10 | 0/10 | Day 34 |
|  | 0/10 | 0/10 | 0/10 | 0/10 | Day 62 |
| Within Normal Limits | 3/10 | 1/10 | 0/10 | 0/10 | Day 6 |
|  | 6/10 | 0/10 | 1/10 | 0/10 | Day 34 |
|  | 4/10 | 1/10 | 2/10 | 0/10 | Day 62 |
| **Gliosis:**  **Thalamus** | **0/10** | **3/10** | **2/10** | **3/10** | **Day 6** |
|  | **0/10** | **1/10** | **2/10** | **5/10** | **Day 34** |
|  | **0/10** | **3/10** | **3/10** | **4/10** | **Day 62** |
| **Gliosis:**  **Cortex** | **4/10** | **3/10** | **3/10** | **3/10** | **Day 6** |
|  | **0/10** | **2/10** | **1/10** | **2/10** | **Day 34** |
|  | **1/10** | **2/10** | **3/10** | **1/10** | **Day 62** |
| Gliosis:  Caudate | 0/10 | 0/10 | 2/10 | 2/10 | Day 6 |
|  | 0/10 | 1/10 | 1/10 | 1/10 | Day 34 |
|  | 0/10 | 0/10 | 1/10 | 0/10 | Day 62 |
| Gliosis:  Hippocampus | 1/10 | 0/10 | 0/10 | 0/10 | Day 6 |
|  | 1/10 | 0/10 | 0/10 | 0/10 | Day 34 |
|  | 0/10 | 1/10 | 2/10 | 1/10 | Day 62 |
| Hemorrhage:  Meninges | 0/10 | 0/10 | 0/10 | 1/10 | Day 6 |
|  | 0/10 | 3/10 | 0/10 | 2/10 | Day 34 |
|  | 0/10 | 2/10 | 0/10 | 1/10 | Day 62 |
| Hemorrhage:  Thalamus | 0/10 | 1/10 | 1/10 | 2/10 | Day 6 |
|  | 0/10 | 0/10 | 0/10 | 0/10 | Day 34 |
|  | 0/10 | 0/10 | 0/10 | 0/10 | Day 62 |
| Hemorrhage:  Cortex | 2/10 | 1/10 | 1/10 | 1/10 | Day 6 |
|  | 0/10 | 0/10 | 0/10 | 0/10 | Day 34 |
|  | 0/10 | 1/10 | 0/10 | 1/10 | Day 62 |
| Hemorrhage:  Caudate | 0/10 | 0/10 | 0/10 | 2/10 | Day 6 |
|  | 0/10 | 0/10 | 0/10 | 0/10 | Day 34 |
|  | 0/10 | 0/10 | 0/10 | 0/10 | Day 62 |
| Hemorrhage:  Hippocampus | 1/10 | 0/10 | 0/10 | 0/10 | Day 6 |
|  | 0/10 | 0/10 | 0/10 | 0/10 | Day 34 |
|  | 0/10 | 0/10 | 0/10 | 0/10 | Day 62 |
| Chronic-Active  Inflammation:  Cortex | 1/10 | 1/10 | 1/10 | 1/10 | Day 6 |
|  | 0/10 | 0/10 | 0/10 | 0/10 | Day 34 |
|  | 0/10 | 0/10 | 0/10 | 0/10 | Day 62 |
| Focal Histiocytic  Inflammation:  Meninges | 0/10 | 0/10 | 0/10 | 1/10 | Day 6 |
|  | 0/10 | 0/10 | 0/10 | 0/10 | Day 34 |
|  | 0/10 | 0/10 | 0/10 | 0/10 | Day 62 |
| Necrosis:  Thalamus | 0/10 | 0/10 | 0/10 | 1/10 | Day 6 |
|  | 0/10 | 0/10 | 0/10 | 0/10 | Day 34 |
|  | 0/10 | 0/10 | 0/10 | 0/10 | Day 62 |
| Necrosis:  Cortex | 0/10 | 0/10 | 2/10 | 0/10 | Day 6 |
|  | 0/10 | 0/10 | 0/10 | 0/10 | Day 34 |
|  | 0/10 | 0/10 | 0/10 | 0/10 | Day 62 |
| Necrosis:  Caudate | 0/10 | 0/10 | 0/10 | 1/10 | Day 6 |
|  | 0/10 | 0/10 | 0/10 | 0/10 | Day 34 |
|  | 0/10 | 0/10 | 0/10 | 0/10 | Day 62 |
| Vacuolization:  Thalamus | 0/10 | 0/10 | 0/10 | 1/10 | Day 6 |
|  | 0/10 | 0/10 | 1/10 | 2/10 | Day 34 |
|  | 0/10 | 0/10 | 0/10 | 1/10 | Day 62 |
| Vacuolization:  Cortex | 0/10 | 1/10 | 0/10 | 0/10 | Day 6 |
|  | 0/10 | 01/10 | 0/10 | 1/10 | Day 34 |
|  | 0/10 | 0/10 | 0/10 | 0/10 | Day 62 |
| Vacuolization:  Caudate | 0/10 | 0/10 | 0/10 | 1/10 | Day 6 |
|  | 0/10 | 0/10 | 1/10 | 1/10 | Day 34 |
|  | 0/10 | 0/10 | 0/10 | 0/10 | Day 62 |
| Clear Space:  Thalamus | 0/10 | 1/10 | 0/10 | 2/10 | Day 6 |
|  | 0/10 | 0/10 | 0/10 | 0/10 | Day 34 |
|  | 0/10 | 0/10 | 0/10 | 0/10 | Day 62 |
| Clear Space:  Cortex | 1/10 | 2/10 | 1/10 | 0/10 | Day 6 |
|  | 0/10 | 0/10 | 0/10 | 1/10 | Day 34 |
|  | 0/10 | 0/10 | 0/10 | 0/10 | Day 62 |
| Clear Space:  Caudate | 0/10 | 0/10 | 0/10 | 01/10 | Day 6 |
|  | 0/10 | 0/10 | 0/10 | 0/10 | Day 34 |
|  | 0/10 | 0/10 | 0/10 | 0/10 | Day 62 |
| Mineralization | 2/10 | 4/10 | 1/10 | 0/10 | Day 6 |
|  | 3/10 | 4/10 | 3/10 | 1/10 | Day 34 |
|  | 4/10 | 3/10 | 1/10 | 5/10 | Day 62 |
| Inclusion Body:  Intranuclear | 0/10 | 0/10 | 0/10 | 3/10 | Day 6 |
|  | 0/10 | 0/10 | 0/10 | 0/10 | Day 34 |
|  | 0/10 | 0/10 | 0/10 | 0/10 | Day 62 |

**Legend:** Hamsters were treated with CRAd-S-pk7 adenoviral vector delivery and were sacrificed at 6, 34, and 62 days post viral vector injection for microscopic pathology observation. Analysis of adverse events associated with vector treatment in the target organs (CNS) is shown above; other organs were analyzed but not included due to lack of any significant viral vector-associated pathology. Analysis shows mild perivascular/meningeal inflammation and mild gliosis in the thalamus/cortex (in bold and shown in Figure 7), which is largely resolved by day 62 after treatment. N=10 hamsters per group/per time point (n=5 male + n=5 female).
